# Retraction: Muminov, A. et al. Modern Virtual Fencing Application: Monitoring and Controlling Behavior of Goats Using GPS Collars and Warning Signals. *Sensors* 2019, *19*, 1598

**DOI:** 10.3390/s20071988

**Published:** 2020-04-02

**Authors:** Sensors Editorial Office

**Affiliations:** MDPI, St. Alban-Anlage 66, 4052 Basel, Switzerland; sensors@mdpi.com

It has come to our attention that the majority of the content in the title paper [1] was copied from the Marini et al. [2] without citation. The editorial board and editorial office consider the concerns sufficiently serious that we have decided to retract the paper. 

MDPI is a member of the Committee on Publication Ethics and takes the responsibility to enforce strict ethical policies and standards very seriously. To ensure the addition of only high-quality scientific works to the field of scholarly publication, [1] is retracted and shall be marked accordingly. We apologize to the readership of *Sensors* for any inconvenience caused. 
